# Marine Algal Polyphenols as Skin Protective Agents: Current Status and Future Prospectives

**DOI:** 10.3390/md21050285

**Published:** 2023-04-30

**Authors:** H.H.A.C.K. Jayawardhana, Thilina U. Jayawardena, K.K.A. Sanjeewa, N.M. Liyanage, D.P. Nagahawatta, Hyo-Geun Lee, Jae-Il Kim, You-Jin Jeon

**Affiliations:** 1 Department of Marine Life Sciences, Jeju National University, Jeju 690-756, Republic of Koreahyogeunlee92@jejunu.ac.kr (H.-G.L.); 2 Department of Chemistry, Biochemistry and Physics, Université du Québec à Trois-Rivières, Trois-Rivières, QC G8Z 4M3, Canada; thilina.uduwaka.jayawardena@uqtr.ca; 3 Faculty of Technology, University of Sri Jayewardenepura, Nugegoda 10250, Sri Lanka; asankasanjeewa@sjp.ac.lk; 4 Department of Food Science and Nutrition, Pukyong National University, Busan 608-737, Republic of Korea

**Keywords:** seaweeds, polyphenol, cosmeceutical, skin

## Abstract

The skin is the outermost anatomical barrier, which plays a vital role in the maintenance of internal homeostasis and protection against physical, chemical, and biological detractors. Direct contact with various stimuli leads to several physiological changes that are ultimately important for the growth of the cosmetic industry. Due to the consequences of using synthetic compounds in skincare and cosmeceutical-related industries, the pharmaceutical and scientific communities have recently shifted their focus to natural ingredients. The nutrient-rich value of algae, which are some of the most interesting organisms in marine ecosystems, has attracted attention. Secondary metabolites isolated from seaweeds are potential candidates for a wide range of economic applications, including food, pharmaceuticals, and cosmetics. An increasing number of studies have focused on polyphenol compounds owing to their promising biological activities against oxidation, inflammation, allergies, cancers, melanogenesis, aging, and wrinkles. This review summarizes the potential evidence of the beneficial properties and future perspectives of using marine macroalgae-derived polyphenolic compounds for advancing the cosmetic industry.

## 1. Introduction

### 1.1. Cosmetic Industrial Background

The use of cosmetics, perfumes, and personal care products dates back to ancient times, dating back to the Egyptian, Greek, and Roman eras. The cosmeceutical sector grows globally every year due to the intensifying modern beauty trends. As mentioned by Kumar (2005), the overall skincare sector accounts for 25% of the USD 124 billion global cosmetic and toiletries market [1]. Cosmeceutical ingredients represent both cosmetics and pharmaceuticals, which are used to improve appearance while improving human skin health [2,3]. Today, the cosmeceutical industry is one of the fastest-growing and most profitable industry in the world. To meet consumer demand, cosmetic industries are shifting to the overuse of synthetic cosmetic ingredients in formulations, such as hydroquinone, benzophenones, dibenzoylmethane, para-aminobenzoic acid, butylhydroxyanisole, and butylhydroxytoluene [4]. The excessive use of synthetic ingredients in cosmeceuticals can lead to various types of toxicities. Over the past few years, consumers have shifted their preference towards natural skincare products to avoid those consequences. Therefore, after the 19th century, chemicals were used to replace more expensive natural ingredients, making cosmetics more widely used [1]. Various natural resources, such as terrestrial plants, fungi, algae, bacteria, and animal products, can be used in the cosmetic industry. Marine resources including macro and microalgae, different fish species, corals, crustaceans, and marine bacteria represent a wide range of valuable compounds with potential applications in nutraceuticals and cosmetics [5,6]. Macro- and microalgae provide vitamins, minerals, proteins, amino acids, sugars, lipids, terpenoids, polyphenolic compounds, pigments, and enzymes that have been considered for their potential applications in cosmetics and cosmeceuticals. In addition, fish is an excellent source of gelatin and collagen; corals and crustaceans can provide chitin, chitosan, astaxanthin, sea mud, and sea salt, which have cosmetic uses [5,7]. Among them, marine macroalgae are currently widely used to improve skin conditions.

### 1.2. Introduction to Marine Algal Polyphenols

Seaweeds, also known as macroalgae, are a widespread group of macroscopic, multicellular organisms that include thousands of species in marine ecosystems. Algae are among the major biomass producers in marine environments and play a vital role in supporting marine biodiversity [8,9]. Based on their cell structure and composition, marine algae may be prokaryotic and eukaryotic organisms [10,11]. Eukaryotic algae can be divided into three main subgroups depending on their color, which is determined by the presence of pigments. Chlorophyll imparts the color green to Chlorophyta (green algae), Phycobilin imparts the color red to Rhodophyta (red algae), and Fucoxanthin imparts the color brown to Phaeophyceae (brown algae) [12,13,14]. Yet, another type of alga is the prokaryotic Cyanobacteria (blue-green alga).

According to archaeological evidence and early written accounts, many cultures have incorporated seaweeds into their diet owing to their functional benefits [15,16]. The demand for algal products has increased in the past decades due to the presence of bioactive compounds or phytochemicals that benefit human well-being in addition to providing basic nutrition [16]. Several investigations on seaweeds have focused on incorporating these natural substances into commercial products [17]. Seaweeds are a good source of nutrients, including carbohydrates, peptides, proteins, vitamins (such as vitamin A, C, D, E, B-complex, and B12) [18], fats, oils, polyunsaturated fatty acids, dietary fibers, and minerals [13,19,20]. In addition to these nutrients, seaweeds contain a high proportion of bioactive compounds, predominantly secondary metabolites, such as polyphenols [17]. The components can vary with the type of seaweed. Table 1 showed different components found in Chlorophyta, Rhodophyta, and Phaeophyceae. These bioactive compounds have commercial applications in the cosmeceutical, nutraceutical, pharmaceutical, medicinal, and agricultural industries [10]. However, novel potential extents need to be explored to maximize the efficient utilization of seaweeds.

Polyphenols are some of the most common groups of substances in the Plantae and Chromista Kingdoms. Polyphenolic compounds extracted from different algal species have a wide range of biological activities, including antioxidant [21,22,23], anti-inflammatory [24], anti-carcinogenic [25,26], anti-diabetic [27,28,29], cardiovascular disorder inhibitory, antihypertensive [13], antiviral [30], and anti-allergic properties [31]. In addition to their role in the inhibition of melanogenesis [32,33,34], elastase and collagenase enzymes [35], matrix metalloproteinases (MMPs), and hyaluronidase [36] are of great importance in cosmeceutical product development. Owing to their wide range of application, polyphenols extracted from marine algae are economically valuable candidates for the mass production of different products. This review focuses on the skin-protective properties of marine algal polyphenols. 

A summary of marine algal polyphenols and their possible cosmeceutical and skincare activities are presented in Table 2.

**Table 1:** Different components in algae.

Constituents	Chlorophyta	Rhodophyta	Phaeophyceae	References
Polysaccharide	Ulvans	Carrageenans	Alginate and Fucan	[37,38]
Lipid and fatty acids	Hexadecatetraenoic, oleic, Palmitic acids, and PUFAs (linoleic acid and α-linolenic acid)	Eicosapentaenoic acid (EPA) and Arachidonic acid (AA)	EPA and AA	[38,39]
Sterol	Ucocholesterol, Cholesterol, and ß-sitosterol	Desmosterol, Cholesterol, Sitosterol, Fucosterol, and Chalinasterol	Fucosterol, Cholesterol, and Brassicasterol	[40,41]
Pigment	Chlorophyll	Phycobilin	Fucoxanthin	[12,13]
Phenolic compounds			phlorotannins (phloroglucinol, eckol, 7-phloroeckol, 6,6-bieckol, phlorofucofuroeckol A, fucodiphloroethol)	[42,43]
	hydroxybenzoic acid derivatives (gallic, p-hydroxybenzoic, vanillic, and syringic acids), hydroxycinnamic acids (caffeic, ferulic, sinapic, and p-coumaric acids), flavonoids (epicatechin, epigallocatechin, rutin, quercitrin, hesperidin, myricetin, and kaempferol), and bromophenols			

**Table 2:** Summary of the bioactive properties of polyphenols isolated from marine macroalgae.

Seaweeds	Activity Potential	Active Component	References
Brown algae			
*Gongolaria nodicaulis* (formerly *Cystoseira nodicaulis*), *Ericaria selaginoides* (formerly *Cystoseira tamariscifolia*), *Gongolaria usneoides* (formerly *Cystoseira usneoides*), *Fucus spiralis*	Anti-aging, antioxidant, anti-wrinkling (inhibition of hyaluronidase), anti-inflammation	Plorotannins-Fucophloroethol, fucodiphloroethol, fucotriphloroethol, 7-phloroeckol, phlorofucofuroeckol and bieckol/diecko	[44]
*Ecklonia cava*	Anti-allergic	Plorotannins-dieckol and 6,6-bieckol	[45]
*Ecklonia cava*	Inhibition of tyrosinase and melanin synthesis	Phlorotannins (ethanolic extract and ethyl acetate soluble fraction)	[46]
*Ecklonia cava*	Inhibition of tyrosinase and melanin synthesis	Phlorotaninns-phloroglucinol, eckol, and dieckol	[47]
*Ecklonia cava*	Anti-inflammation, inhibited MMP expression	Phloroglucinol derivatives	[48]
*Ecklonia cava*	Antioxidant, anti-photoaging	100% methanol extract–phlorotannins (dieckol)	[49]
*Ecklonia cava*	Antioxidant, anti-inflammation, anti-photoaging	Dieckol	[50]
*Eisenia arborea* *Sargassum thunbergii*	Anti-allergic	Polyphenolic compounds (Methanol extract)	[51]
*Eisenia arborea*	Anti-allergic	phlorofucofuroeckol-B	[52]
*Eisenia bicyclis* *Ecklonia cava* *Ecklonia cava* subsp. *stolonifera* (formerly *Ecklonia stolonifera*)	Anti-photoaging	Phlorotannins- eckol and dieckol	[53]
*Eisenia bicyclis* *Ecklonia cava* subsp. *kurome* (formerly *Ecklonia kurome*)	Anti-wrinkling (inhibition of hyaluronidase)	Phlorotannins- phloroglucinol, eckol, phlorofucofuroeckol A, dieckol, and 8,8- bieckol	[54]
*Ecklonia cava* subsp. *stolonifera* (formerly *Ecklonia stolonifera*)	Inhibition of tyrosinase	phloroglucinol, eckstolonol, eckol, phlorofucofuroeckol A, and dieckol	[55]
*Ishige okamurae*	Inhibition of tyrosinase, antioxidant, whitening	Phenolic compounds (methanol extract)	[56]
*Sargassum longifolium*	Antioxidant, anti-bacterial	Phenolic compounds (ethanol extract)	[57]
*Eisenia bicyclis* *Ecklonia cava* *Ecklonia cava* subsp. *kurome* (formerly *Ecklonia kurome*)	Antioxidant	Phloroglucinol, Eckol, Phlorofucofuroeckol A, Dieckol and 8,8′-bieckol	[58]
*Sargassum carpophyllum*	Anti-allergic	Phlorotannins – 2-[2-(3,5-dihydroxyphenoxy)-3,5-dihydroxyphenoxy]-1,3,5-benzenetriol 2,2′-[[2-(3,5-dihydroxyphenoxy)-5-hydroxy-1,3-phenylene]bis(oxy)]bis(1,3,5-benzenetriol 2-[2-[4-[2-(3,5-dihydroxyphenoxy)-3,5-dihydroxyphenoxy]-3,5-dihydroxyphenoxy]-3,5-dihydroxyphenoxy]-1,3,5-benzenetriol	[59]
*Colpmenia sinuosa*	Anti-inflammation, antimicrobial	Ethanolic and dichloromethane extract respectively	[60]
Green algae			
*Dunaliella tertiolecta* (green microalga) *Tetraselmis* (green microalga) *Nannochloropsis* sp. (Eustigmatophyceae)	Anti-aging	Phenolic compounds (Ethanolic extract)	[61]
*Turbinaria conoides*	Antioxidant	Phenolic compounds (ethanolic extract and ethyl acetate)	[62]
*Ulva lactuca*	Antioxidant	80% methanolic extract	[63]
*Ulva linza*	Anti-inflammation, antimicrobial	Ethanolic extract	[60]
*Caulerpa racemosa*	Antioxidant, anticancer	clionasterol-rich hexane fraction	[64]
*Ulva compressa* (formerly *Enteromorpha compressa*) *Capsosiphon fulvescens* *Chaetomorpha moniligera* *Ulva australis* (formerly *Ulva pertusa*)	Antioxidant	Ethanolic extract	[65]
Red algae			
*Acanthophora spicifera*	Antioxidant	Flavonoids-acanthophorin A, acanthophorin B, tilimside, (-)-catechin, quercetin	[66]
*Vertebrata fucoides* (formerly *Polysiphonia fucoides*)	Antioxidant	Phenolic compounds (ethanolic extract)	[67]
*Corallina pilulifera*	Antioxidant, anti-photoaging	Phenolic compounds (methanol extract)	[68]
*Gracilaria foliifera*	Antioxidant, anti-bacterial	Phenolic compounds (ethanol extract)	[57]
*Kappaphycopsis cottonii* (formerly *Eucheuma cottonii*)	Antioxidant	Phenolic compounds (ethanolic extract and ethyl acetate)	[62]
*Hypnea musciformis* *Hypnea valentiae* *Jania rubens*	Antioxidant	Methanol extract and its solvent fractions (n-hexane, dichloromethane, and ethyl acetate)	[69]

### 1.3. Classification of Marine Algal Polyphenols 

Polyphenol families can be classified according to their biological functions, sources of origin, and chemical structures. Polyphenols can be categorized into two main subfamilies, flavonoids and nonflavonoids, based on the number of phenol rings and the structural elements that bind these rings to one another [70]. Flavonoids consist of a common C6-C3-C6 skeleton with two phenyl rings that are linked by a heterocyclic ring. Non-flavonoids consist of common C6-C2 non-flavonoid compounds, which can be divided into phenolic acids, stilbenes, and lignans [71,72]. 

**Figure 1:**
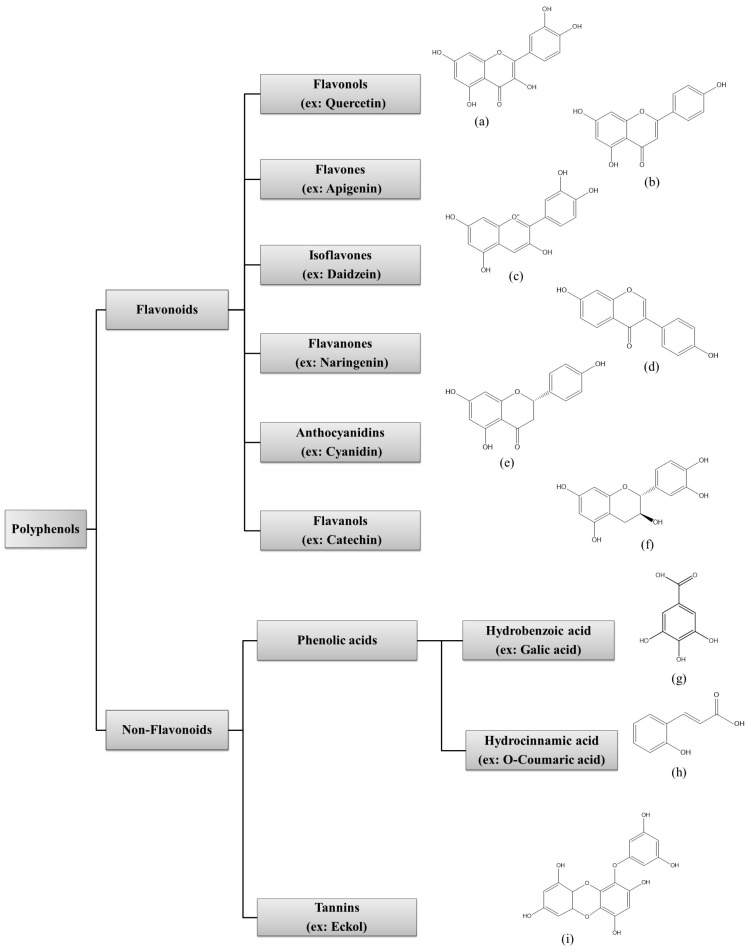
Classification of marine algal polyphenols according to chemical structure: (**a**) Quercetin; (**b**) Apigenin; (**c**) Daidzein; (**d**) Naringenin; (**e**) Cyanidin; (**f**) Catechin; (**g**) Galic acid; (**h**) O-Coumaric acid; (**i**) Eckol.

Flavonoids are the most extensively distributed subfamily and can be further divided into six subclasses: flavones, isoflavones, flavonols, flavanols, flavanones, and anthocyanins (Figure 1) [13,73]. These polyphenolic compounds can be isolated from different algal species. Some examples for each subfamily are mentioned in Table 3. Tannins are a family of phenolic metabolites, which have distinct properties, such as the ability to bind to proteins, large molecular-size compounds, pigments, and metallic ions [22]. In recent decades, they have received considerable attention owing to their antioxidant capacity [74]. Phenolic acids possess a phenol moiety and a resonance-stabilized structure with the ability to donate protons, resulting in antioxidant activity through radical scavenging mechanisms. Furthermore, phenolic acids exhibit other health-protective effects, such as antimicrobial, anti-inflammatory, anticancer, and anti-mutagenic effects [75]. Stilbenes and lignans are small groups widely distributed in the plant and Chromista kingdoms, and can be identified as protectors against protozoans, viral diseases, and some forms of cancer [76].

Polyphenolic compounds can be found in different marine organisms, which include macroalgae, microalgae, cyanobacteria, fungi, seagrasses, and sponges [77]. Among these organisms, marine algal species accumulate large amounts of polyphenolic compounds, including, in particular, phlorotannin, phloroglucinol and its polymers [78]. Algal species may differ from one another regarding the polyphenols produced. Bromophenols, phenolic acids, and flavonoids are common polyphenolic compounds in green and red algae, whereas phlorotannin is dominant in marine brown algae and can be categorized as a group of complex polymers of phloroglucinol (1,3,5-trihydroxybenzene) [13].

**Table 3:** Polyphenolic compounds isolated from marine algae.

	Isolated Compound	Seaweed	Reference
Flavonols	Quercetin	*Acanthophora spicifera* *Caulerpa racemosa*, *Caulerpa racemosa*, *Caulerpa racemosa*, *Caulerpa scalpelliformis*, *Codium dwarkense*, *Ulva fasciata*, and *Ulva lactuca* (formerly *Ulva fasciat*) (Chlorophyta)	[66,79]
Flavones	Apigenin	*Acanthophora spicifera* *Caulerpa racemosa*, *Caulerpa racemosa*, *Caulerpa racemosa*, *Caulerpa scalpelliformis*, *Codium dwarkense*, *Ulva fasciata*, and *Ulva lactuca*	[79,80]
Isoflavones	Daidzein	*Sargassum muticum*, *Sargassum vulgare* (Phaeophyceae), *Hypnea spinella*, *Porphyra* sp. (Rhodophyta), *Undaria pinnatifida* (Phaeophyceae), *Chondrus crispus* and *Halopytis incurvus* (Rhodophyta)	[81]
Flavanones	Naringenin	*Undaria pinnatifida*	[82]
Anthocyanidins	Cyanidin	*Caulerpa racemosa*, *Caulerpa racemosa*, *Caulerpa racemosa*, *Caulerpa scalpelliformis*, *Codium dwarkense*, *Ulva fasciata*, and *Ulva lactuca*	[79]
Flavanols	Catechin	*Acanthophora spicifera*	[66]
Hydrobenzoic acid	Gallic acid	*Laminaria digitata*, *Dictyota dichotoma*, *Fucus vesiculosus*, *Fucus serratus*, *Fucus distichus*, *Fucus spiralis*, *Mastocarpus stellatus*, *Vertebrata fucoides* (formerly *Polysiphonia fucoides*) (Rhodophyta), *Saccharina latissima*, *Gracilaria vermiculophyllum* (Rhodophyta), *Palmaria palmata*, *Porphyra purpurea*, *Chondrus crispus*, *Ulva intestinalis* (formerly *Enteromorpha intestinalis*) (Chlorophyta), *Ulva lactuca*, *Sargassum muticum*	[67]
Hydrocinnamic acid	Coumaric acid	Kelp *Jania rubens*	[83,84]
Tannins	Eckol	*Eisenia bicyclis*, *Ecklonia cava* subsp. *kurome* (formerly *Ecklonia kurome*) (Phaeophyceae) *Eisenia bicyclis*, *Ecklonia cava*, *Ecklonia cava* subsp. *Kurome* (formerly *Ecklonia kurome*) (Phaeophyceae)	[54]

### 1.4. Skin Protective Properties

Human skin is the outermost anatomical barrier, which acts as a fence that protects the internal organs of the body against physical, chemical, and biological detractors [85,86]. Chronic exposure to environmental stressors triggers a series of biological outcomes, including the generation of reactive oxygen species (ROS) and inflammatory responses, and the expression of matrix metalloproteinases, all of which impair skin functions and repair capacity, turning it thinner and more fragile while gradually losing its natural elasticity and altering its hydration process [87]. Consequently, the skin continuously experiences aging [88]. 

Normally, sun exposure results in irregular skin pigmentation and a coarse and rough appearance, with deep lines and wrinkles [89]. In addition, cellulitis, dermatitis, rashes, acne, and other skin ailments are skin-related problems that humans face in their day-to-day lives. Thus, skincare products are manufactured due to the consumer demand for products that help overcome these problems [90]. Recent developments in marine biotechnology offer a great opportunity to study inflammation, skin aging, and skin degradation. Many dermatological studies have suggested that marine bioactive compounds can positively affect the treatment of skin disorders [14]. According to a study on the global status of seaweed production by Ferdouse et al., 40% of the world’s hydrocolloid market has witnessed the importance of seaweeds in the cosmeceutical industry [91].

## 2. Marine Algal Polyphenols as Skin Protective Agents

**Figure 2:**
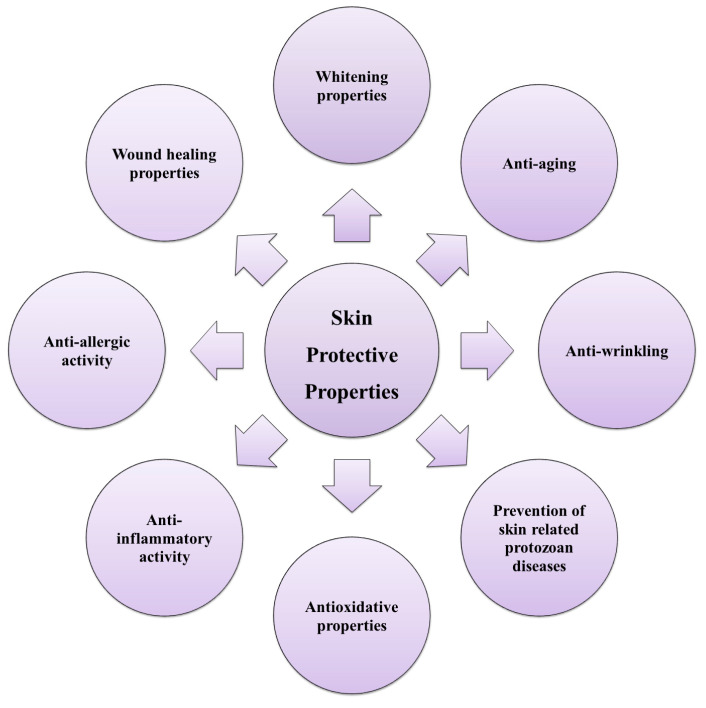
Skin protective properties of marine algal polyphenols.

Marine algal polyphenols exhibit different skin protective properties, as illustrated in Figure 2.

### 2.1. Antioxidative Activity (ROS Scavenging Activity)

Based on the mechanism of antioxidant activity, a compound can be classified as a radical scavenger or an oxygen scavenger. Among these two mechanisms, radical scavenging is the most common and important method used to determine antioxidant activity [92]. When human skin is exposed to environmental stresses, such as pollutants and ultraviolet radiation (UV), it induces the production of ROS, which subsequently generates oxidative stress [93]. ROS are a cluster of highly reactive compounds that show high reactivity against essential biomolecules owing to the presence of free radicals. Therefore, it is essential to identify antioxidative agents that can be effectively used against free radicals. Generally, seaweeds are continuously exposed to extreme environmental conditions that induce the accumulation of polyphenols to fight stress, suppress the oxidation process, scavenge ROS, and reduce DNA damage [94]. Thus, this literature review includes bioactive compounds isolated from seaweeds, especially polyphenolic compounds, which can play a vital role in the pharmaceutical and cosmeceutical industries as photo protectors owing to their high potential for antioxidative activity [92,95,96].

Farvin and Jacobsen (2013) reported that ethanolic extracts of *Vertebrata fucoides* (formerly *Polysiphonia fucoides*) and *Fucus* spp. (Phaeophyceae) collected along coasts in Denmark showed the best antioxidant activity among 16 different algal species. Antioxidant activity was measured using four in vitro antioxidant assays, including 2,2-diphenyl-1-picrylhydrazyl (DPPH) radical scavenging activity (RSA), reducing power, ferrous ion-chelation, and inhibition of oxidation on the liposome model system [67]. Devi et al. (2008) studied the in vitro antioxidant activity of 10 different algal species collected from Tamil Nadu, India. *Gelidiella acerosa*, which belongs to the phylum Rhodophyta, possesses high antioxidant activity that might help prevent or decelerate the progression of various oxidative stress-related disorders [97]. According to the investigation conducted by Shibata et al. (2007), polyphenols, such as phloroglucinol (1,3,5-trihydroxybenzene), eckol, phlorofucofuroeckol A, dieckol, and 8,8′-bieckol, isolated from *Eisenia bicyclis*, *Ecklonia cava*, and *Ecklonia cava* subsp. *Kurome* (formerly *Ecklonia kurome*) (brown algae), showed significant RSA against DPPH (50% effective concentration values: 12–26 μM) and the superoxide anion (50% effective concentration values: 6.5–8.4 μM) and were more effective than α-tocopherol and ascorbic acid [58]. 

Another study reported that dieckol isolated from *Ecklonia cava* is a potent source as a photoprotective agent, which showed a significant protective effect against UVB-induced skin damage in human dermal fibroblasts. The results indicated that dieckol effectively reduced the intracellular ROS activity while improving cell viability, which proved to be a potent ingredient in the cosmeceutical industry [50]. Another study confirmed that fucoxanthin isolated from *Sargassum siliquastrum* (Phaeophyceae) has the ability to protect against oxidative stress induced by UV-B radiation [98].

Airborne particulate matter (PM) is considered an environmental contaminant that has become a global concern due to the oxidative stress that leads to apoptosis and skin damage. A previous studies has reported the protective effect of *Caulerpa racemose* (Chlorophyta), clionasterol-rich hexane fraction (CRHF) against PM-induced skin damage using human keratinocytes and a zebrafish model. The results revealed that the sample exhibited a superior protective effect via downregulating intracellular ROS levels and mitochondrial ROS levels. Additionally, in vivo results showed that CRHF significantly downregulated the PM-induced cell death, NO production, and ROS levels in the zebrafish model [64].

Hence, these results evidenced that phenol-rich extracts and phenolic compounds are potent sources of antioxidative agents. Moreover, research findings related to polyphenols isolated from different algae and their antioxidant properties are summarized in Table 1. 

### 2.2. Anti-Inflammatory and Anti-Allergic Activity

Inflammation is a key aspect of the host response and may be caused by various stimuli, including physical damage, ultraviolet irradiation, microbial invasion, and immune reactions. Redness, warmness, swelling, and pain are the typical features of inflammation. Prolonged inflammation is harmful to humans and contributes to the pathogenesis of many diseases, including cancer, chronic asthma, multiple sclerosis, and psoriasis [99]. Inflammation is divided into acute and chronic, with the acute phase involving fluid accumulation and increased blood flow, leukocyte and vascular permeability, while the chronic phase is associated with the initiation of an immune response [100]. Furthermore, exposure to the UV radiation stimulates different inflammatory responses in human skin, including the transendothelial migration of leukocyte at the injured site, the escape of plasma protein from the bloodstream, microvascular structural changes, and vasodilation [101].

UV radiation can cause inflammation by altering chemical reactions in the skin via various mediators, such as nitric oxide (NO), inducible NO synthase, prostaglandin E2, cyclooxygenase-2 (COX-2), tumor necrosis factor-α, and other cytokines, such as interleukin 1 and interleukin 6, which are regulated by nuclear factor kappa B (NF-κB) in keratinocytes. NF-κB is associated with skin diseases such as psoriasis, dermatitis vulgaris and allergic dermatitis, which cause skin dehydration, irritation, swelling, itching, redness, and rash in the affected areas, and also induce MMP-1 expression, which leads to aging [102]. 

Fine dust particles have become a major cause of air pollution, which results in strong inflammatory responses in human skin. Fernando et al. (2017) reported on the anti-inflammatory potential of diphlorethohydroxycarmalol (DPHC) isolated from *Ishige okamurae* (Phaeophyceae) against fine-dust-induced inflammation [103].

The keratinocyte model is commonly used to evaluate the effect of irritants as these cells are located in the outer layers of the skin. Keratinocytes have the ability to produce a broad range of inflammatory mediators that induce the upregulation and the secretion of secondary mediators, such as chemokines, which results in the recruitment of leukocytes to the damaged area of the skin [103]. Environmental irritants that cause skin inflammation include contact allergens and ultraviolet light, which trigger the cutaneous inflammatory response by directly inducing epidermal keratinocytes to produce specific pro-inflammatory cytokines and adhesion molecules. Keratinocytes can act as “signal transducers”, capable of converting exogenous stimuli into the production of cytokines, adhesion molecules, and chemotactic factors, which are responsible for initiating “antigen-independent” skin inflammation. The initiation phase may facilitate or promote the amplification phase with additional production of tumor necrosis factor alpha (TNF-α) and interferon gamma (IFN-γ) through an “antigen dependent” pathway and keratinocyte/T cell/antigen presenting dendritic cell associations. The direct activation of keratinocytes, with their ability to produce a full range of pro-inflammatory cytokines, can strongly influence endogenous and recruited immunocompetent cells, thus providing a critical trigger responsible for rapid and clinical alterations [104].

An allergy is a hypersensitivity reaction of the immune system that occurs due to harmful environmental stimuli, such as food, pollen, insects, mites, animal dander, and chemical agents. At present, different types of drugs belonging to several classes, such as antihistamines, nonsteroidal anti-inflammatory drugs, corticosteroids, and aspirin, are used against inflammatory and allergic disorders. However, long-term use and high-dose medications may lead to substantial side effects. Therefore, it is essential to improve the tolerability of these drugs for long-term satisfactory usage [24]. Many studies have shown that natural polyphenols can be effectively used for allergy remission and inflammatory disorders [105]. 

Atopic dermatitis (AD) is a challenging skin inflammatory disease that has an allergic form, characterized by elevated eosinophilia and serum immunoglobulin E (IgE) levels, and positive skin prick test reactions to common environmental allergens, such as food or aeroallergens [53,106]. Currently, a better therapeutic approach is essential because general treatments are much more expensive, and every age stage is susceptible to skin inflammatory diseases. Therefore, research is ongoing to screen for bioactive substances from natural resources that have the potential to inhibit the production of IgE. Sugiura et al. (2006) reported that phlorofucofuroeckol-B isolated from *Eisenia arborea* (Phaeophyceae) exhibited anti-allergic activity in rat basophilic leukemia (RBL)-2 H3 cells. The IC_50_ value obtained for the positive control (epigallocatechin gallate) and the sample (phlorofucofuroeckol-B) were 7.8 and 22 μM, respectively. Hence, this study suggested that phlorofucofuroeckol-B can be used as a potent anti-inflammatory agent against AD [52]. Several studies proved that polyphenolic-compound-rich extracts from brown algae, specially *Sargassum* sp. [107], *Ecklonia* sp., and *Eisenia* sp. [108] have significant activity against inflammation and AD. Recently, a number of brown algae have been recognized for their ability to fight allergic responses compared to other groups of algae, as fewer studies the red and the green algae were conducted [31].

### 2.3. Wound Healing of the Skin

A wound is a damage or disruption of the normal anatomical structure and function of skin, which can range from a simple break in the epithelial integrity of the skin or can be deeper, extending into the subcutaneous tissue, and damaging other structures, such as tendons, muscles, vessels, nerves, parenchymal organs, and even bones [109]. Generally, wound healing is a dynamic and complex process involving cellular, molecular, biochemical, and physiological activities that result in the regeneration and replacement of injured connective tissue at the wound site [109,110]. Since ancient times, plant materials have been used to accelerate the wound-healing process. Plants are believed to have natural therapeutic effects against endocrine disorders and other diseases. They can also be used to treat injuries, such as cuts or burns, using simple formulation procedures, making them ideal candidates for natural remedies [110]. Plant-based products contain active ingredients, plant parts or combinations. Before the advent of modern medicine, people have used medicines of plant origin. It is known that ancient Egyptian medicine from 1000 B.C. used garlic, opium, castor oil, coriander, mint, and indigo plants to accelerate the wound healing process. Furthermore, Indian Ayurvedic medicine has used many herbs, such as turmeric, as early as 1900 B.C [111]. Aloe vera is one of the examples that has been used for many centuries and is the major ingredient in various commercial skin and wound-care products, especially for burn wounds. Much scientific research evidences that aloe vera can improve wound healing rates [112]. Additionally, terrestrial plants, such as *Aspilia Africana*, showed inhibition of microbial growth, accelerated healing; methanol extracts of *Heliotropium indicum* leaves improve the wound healing rate; *Elaeis guineensis* leaf extract improves the tissue generation [111].

Currently, researchers are targeting algal species owing to their promising wound-healing properties, especially for treating diabetes mellitus patients. *Spirulina platensis* is a blue-green alga that has been tested to examine the wound healing efficiency of various solvent extracts (50 μg/ml of methanolic, ethanolic, and aqueous extracts) using an in vitro scratch assay on human dermal fibroblast cells (HDF). Although methanolic and ethanolic extracts did not show better results than aqueous extracts, they did exhibit a proliferative effect [93]. The ethanolic extract of *Kappaphycopsis cottonii* (formerly *Eucheuma cottonii*) (Rhodophyta) increased the rate of wound contraction and epithelization compared to the positive and negative control groups. The ethanolic extract was approximately 20% more effective than the aqueous extract. In vivo experiments treated with ethanolic extract, aqueous extract, and the positive control (honey) showed 100%, 83.44%, and 93.76% inhibition, respectively, compared to the control (52.66 %). Fard et al. (2011) reported that *K. cottonii* ethanolic extract is effective for wound healing within the shortest possible period [113].

The wound healing process is always associated with wound treatments that improve and guide the healing process. The usual care routine includes taking a swab for infection, cleaning the wound bed, and finally, dressing the wound. The ideal wound dressing should promote rapid recovery while minimizing patient discomfort. In addition, it must be able to remove excess exudate, enhance autolytic debridement, and maintain sufficient hydration for healing [114]. Wound dressings should be flexible, sticky, and easy to remove. Currently, both natural and synthetic polymers are used for the production of artificial dressings. However, the biocompatibility and biodegradability, together with the high degree of biomimesis and physicochemical properties of natural biopolymers, make them particularly interesting for use as components of wound dressings [115,116]. In addition, these components can potentially act as antimicrobial and anti-inflammatory agents, which speeds up the healing process [117].

According to Kim et al. (2020), polyvinyl alcohol hydrogels (PVA) containing diphlorethohydroxycarmalol (DPHC) from *Ishige okamurae* showed an anti-bacterial effect in wound healing applications. The minimum inhibitory concentration of DPHC against *Staphylococcus aureus* and *Pseudomonas aeruginosa* was recorded as 128 μg/mL and 512 μg/mL, respectively. Furthermore, the proposed PVA/DPHC hydrogels showed a strong wound-healing effect compared to non-treated groups of ICR mice in vivo. Additionally, the microstructural, rheological, swelling, thermogravimetric, drug release, gel fraction, cytotoxicity, histological, and bacteria-killing properties proved the wound healing ability of the proposed PVA/DPHC hydrogels. Hence, the data suggested that PVA/DPHC hydrogels have great potential for use in wound healing [118]. 

Based on the above studies, extracts from marine algae can be considered a potential source of therapeutic agents for wound healing and the associated complications.

### 2.4. Anti-Aging Activity and Anti-Wrinkling Activity

Skin aging is a natural, complex biological process that causes physiological changes in the skin due to extrinsic factors, such as environmental conditions, smoking, exposure to UV radiation, toxins, and other intrinsic factors, including genetic or physiological changes [119,120]. Aging reduces the elasticity and thickness of the skin, resulting in an irregular texture and wrinkle formation [36,119]. In addition, because of the low moisture level, the skin becomes drier with wrinkles, which causes premature aging [93]. Collagen and elastin are the main components of skin support. Hyaluronic acid is one of the major components involved in tissue repair and water retention. The simultaneous breakdown of these components with aging may also cause wrinkles and loss of skin firmness. Moreover, these processes may alter the facial contour [14].

Today, aging has become a social problem as people are more concerned about their outer appearance. Therefore, the global demand for natural skincare products with anti-wrinkling properties has gradually increased. Wrinkles are lines and depressions that form during skin aging. They are folds that appear in the skin over the years due to the movements of limbs and muscles while performing various activities and during facial expressions. When these folds are associated with the effects the sun has on the skin, they may cause changes in skin quality. The areas of the face, such as the forehead, cheeks, corners of the eyes, neck, hands, and arms, are more vulnerable to wrinkle formation. Therefore, wrinkling is an index of an individual’s age [14].

Due to the UVB exposure, MMP expression can be accelerated by the activation of upstream transcription factors, such as activator protein-1 and NF-B p65. On the other hand, the MAPK signaling pathway controls the activation of activator protein-1 and, as a consequence, IκB kinase, phosphoinositide-3-kinase-Akt, and p38 MAPK. Therefore, the aforementioned mediators can sequentially activate NF-κB, causing the production of inflammatory cytokines other than MMP. Thus, the inhibition of the UV-B-induced activation of NF-κB and MAPK pathway proteins is critical for reducing skin photoaging [121].

Compounds with the capability of enhancing the inhibition of enzymes, such as MMP, hyaluronidase, collagenase, and elastase, may have the potential to act as active ingredients in the development of novel anti-wrinkle skincare products [36,44,122]. In addition, moisture retention ability is an important factor in skincare products [93]. Most marine algal polyphenols possess these properties. Several previous studies have examined skin protective properties, including the anti-wrinkle activity, of algal secondary metabolites; however, the use of marine algal polyphenols is yet to be elucidated. Thus, advancements in seaweed compound isolation would be beneficial for the development of the cosmeceutical industry [120].

According to Joe et al. (2006), *Eisenia bicyclis*, *Ecklonia cava*, and *Ecklonia cava* subsp. *stolonifera*, which belong to the family Alariaceae, can inhibit the expression of MMP-1 in human dermal fibroblasts, and eckol and dieckol isolated from *E. cava* subsp. *stolonifera* play a vital role in the inhibition of MMP-1 expression. Thus, eckol and dieckol could be effective as potential therapeutic agents for skin aging [53]. Similarly, the ability of the *Corallina pilulifera* (red algae) methanol extract to inhibit free radical oxidation and reduce the expression of UV-induced MMP-2 and MMP-9 in HDFs indicates that it may be a good source of natural anti-photoaging compounds [68]. One study showed that *Sargassum. tennerimum* (Phaeophyceae) was found to be a strong inhibitor of the hyaluronidase enzyme with a 50% inhibitory concentration (IC_50_) of 21 µg/mL. Meanwhile, disodium cromoglycate, an antiallergic drug, had an IC_50_ value of 39 µg/mL, and catechin, a natural hyaluronidase inhibitor, had an IC_50_ value of 20 µg/mL [31]. Five known phlorotannins, namely diecol, ecol, phloroglucinum, florofucocofuroecol, 8,8′-biecol, and an unknown tetramer isolated from *E. kurome* and *E. bicyclis* were tested in vitro for their ability to inhibit hyaluronidase activity compared to three commercial inhibitors hyaluronidases, including catechin, epigallocatechin gallate, and disodium cromoglycate. These phlorotannins have been proven to have a stronger inhibitory effect on hyaluronidase than catechins and sodium cromoglycate. According to these findings, 8,8′-Biecol showed the highest hyaluronidase inhibition among them with an IC value of 40 μM, which was about fifteen times stronger than catechin and seven times stronger than disodium cromoglycate [54]. According to the published findings, polyphenolic compounds isolated from brown algae showed significantly higher activity compared to other groups. Phlorotannins are exclusively derived from marine brown algae, which contain a molecular structure with numerous phenol rings, which endows them with excellent radical scavenging and antioxidant properties. Several in vitro and in vivo studies were carried out, and the findings confirmed that Phlortannins are potential bioactive compounds with excellent activity against aging and wrinkling [123,124].

### 2.5. Whitening Properties

Most women in Asian countries prefer fairer skin tones. Therefore, skin-whitening products have become best-selling items compared to other skincare products in Asia [125]. Skin whitening can be achieved by inhibiting the transcription factors associated with microphthalmia, interference with melanosome maturation and transfer, and suppression of melanocortin 1 receptor activity and tyrosinase enzyme [126]. Among these, tyrosinase inhibition has become the most popular in the cosmetic industry [127]. The accumulation of ROS enhances tyrosinase activity, which may lead to abnormal melanin production [128]. Melanin is a pigment in mammalian skin that determines skin color and plays an important role in protecting the skin from UV radiation and scavenging chemicals and drugs [36]. Consequently, the overproduction and accumulation of melanin lead to skin disorders such as premature aging, dryness, and hyperpigmentation (dark spots) [129,130,131], as well as melanoma skin cancers. Therefore, the inhibition of tyrosinase significantly promotes skin-lightening activity [93]. 

In detail, the expression of the tyrosinase, tyrosinase-related protein 1 (TYRP1) and dopachrome tautomerase (DCT) genes in melanocytes is driven by microphthalmos-associated transcription factor (MITF). Upon binding the alpha-melanocyte-stimulating hormone (α-MSH) or other pro-opiomelanocortin-derived peptide hormones, the melanocortin 1 receptor (MC1R), a G protein-coupled receptor, undergoes a conformational change to enhance the dissociation of the G protein subunits and activate adenylate cyclase, which produces cyclic AMP (cAMP) [132]. The transcription factor cAMP-dependent protein-binding element (CREB) then becomes active under the action of cAMP-dependent protein kinase A (PKA) and promotes the expression of downstream target genes, including MITF. Gene expression and MITF activation are also regulated by other mechanisms, including the c-kit or WNT pathways. The newly expressed tyrosinase protein then undergoes post-translational modifications to the active mature form [133]. Melanin synthesis begins with the oxidation of L-tyrosine and/or L-3,4-dihydroxyphenylalanine (DOPA) to the quinone L-DOPA, which is catalyzed by the enzyme tyrosinase. These enzymatic reactions are common regulatory points in the biosynthetic pathways, and thus, tyrosinase is considered a useful target for controlling undesirable skin pigmentation. There are several strategies for influencing tyrosinase to control melanin synthesis, including the modulation of the tyrosinase gene expression during transcription and translation, the modulation of the post-translational modifications of the tyrosinase protein and its proteolytic degradation, and the modulation of the tyrosinase catalytic activity [134].

Many synthetic chemicals, such as arbutin, kojic acid, and hydroquinone, have been used as tyrosinase inhibitors or whitening agents for the production of skin-whitening products. However, the use of synthetic chemicals is limited because of their low stability, toxicity, low activity, and poor skin penetration [135]. Therefore, nowadays, researchers have focused on natural compounds because people believe that these compounds are much safer than commercially available synthetic skin whiteners [136].

Among 17 seaweeds, *Ecklonia cava* subsp. *Stolonifera*, which belongs to the Laminariaceae family, showed high tyrosinase inhibitory activity. Phloroglucinol derivatives, such as phloroglucinol, eckstolonol, eckol, phlorofucofuroeckol A, and dieckol, isolated from *Ecklonia cava* subsp. *stolonifera* inhibited the oxidation of L-tyrosine catalyzed by mushroom tyrosinase, with IC_50_ values of 92.8, 126, 33.2, 177, and 2.16 µg/mL, respectively. Furthermore, Kang (2004) revealed that polyphenols isolated from marine algae could be used to control tyrosinase activity inhibition [55]. Paudel et al. (2019) investigated the anti-tyrosinase activity of *Symphyocladia latiuscula* (Harvey) Yamada, which belongs to the family Rhodomelaceae and is a rich source of bromophenols. Among the three different bromophenols obtained using column chromatography, 2,3,6-tribromo-4,5-dihydroxybenzyl methyl alcohol and bis-(2,3,6-tribromo-4,5-dihydroxybenzyl methyl ether) exhibited significant tyrosinase inhibitory activity against l-tyrosine substrates, with IC_50_ values of 10.78 ± 0.19 and 2.92 ± 0.04 μM, respectively. In addition, these compounds showed dose-dependent inhibition of melanin and intracellular tyrosinase levels in α-melanocyte-stimulating hormone (α-MSH)-induced B16F10 melanoma cells [137]. Furthermore, Chan et al. (2011) evaluated the tyrosinase inhibition and melanin synthesis inhibition potential of *Sargassum polycystum* ethanolic extract and their fractions. The results showed that the non-polar hexane fraction significantly inhibits the cellular tyrosinase inhibitory activity [138]. Fucoxanthin, isolated from *Saccharina* japonica (formerly *Laminaria japonica*) (Japanese kombu) (Phaeophyceae), has been reported to inhibit tyrosinase activity in UVB-irradiated guinea pigs and melanogenesis in UVB-irradiated mice. Oral administration of fucoxanthin significantly downregulated skin mRNA expression associated with melanogenesis, suggesting that fucoxanthin negatively regulates the melanogenesis factor at the transcriptional level [139].

The bioactive components isolated from brown algae, which specifically include phlorotannin, as well as fucoidan, exhibit outstanding performance compared to other compounds. In the last two decades, many studies proved the bioactive potential of those compounds by using different species in vitro, as well as in vivo models against melanin production [36,47,55,140,141,142]. Hence, marine algae have become the core of several types of research to explore effective skin-whitening agents.

### 2.6. Anticancer Activity

The skin is the outermost barrier in the body and protects against mechanical damage, detrimental substances, pathological invasion, and radiation. Several factors can lead to the initiation and development of cutaneous alterations. Among these factors, excessive exposure to UV radiation is the most prominent risk factor for skin cancer [143]. There are three broad wavelength UV radiations: UVC (100–280 nm), UVB (280–315 nm), and UVA (315–400 nm). UVB alters the structure and function of the dermal and epidermal layers. UVB is also the main factor involved in the initiation and development of melanoma and non-melanoma skin cancers [144]. 

Some epidemiological studies have revealed that skin cancer, which occurs due to mutations in cancer-related genes, is the most dominant type of cancer worldwide. The continuous growth of skin cancer may significantly affect the global health system, as well as the economy [145]. Damage to cells by UVB radiation occurs through the production of ROS, increasing cellular oxidative stress, which ultimately leads to apoptosis. As reported in many previous studies, apoptosis induced by oxidative stress mainly proceeds along the path of mitochondria-mediated apoptosis. Events are initiated by the permeabilization of the outer mitochondrial membrane, which causes the release of apoptogenic factors, such as endonuclease G, cytochrome c, and apoptosis, inducing factor (AIF), present in the mitochondrial intermembrane space. Caspase-mediated apoptosis pathways are further exacerbated by p53 activation, which initiates the expression of pro-apoptotic factors. Effector caspases cause cleavage of the nuclear enzyme PARP, one of the most important mediators of DNA stability, repair, and transcription [146]. In general, chemotherapy, radiotherapy, immunotherapy, and targeted therapy are used as treatments but are highly toxic, expensive, and sometimes ineffective [147]. Therefore, it is essential to investigate a novel and effective method to treat patients with cancer.

In the last few years, algae have been used as sources of bioactive molecules with potential activity against cancer and inflammation [101]. Several studies have revealed that marine algal polyphenols have potential anticancer activity. Hwang et al. demonstrated that polyphenols extracted from brown algae effectively suppressed UVB-induced skin carcinogenesis in the SKH-1 hairless mouse skin model [148]. In in vitro experiments performed on *Kappaphycopsis cottonii*, edible red algae had an anti-proliferative activity on estrogen-dependent MCF-7 and estrogen-independent MB-MDA-231 human breast cancer cells at concentrations of 20 and 42 µg/mL, respectively, owing to their high phenolic content [149]. Furthermore, some in vivo studies confirmed that brown algal polyphenols can suppress COX-2 expression and cell proliferation [150]. These results suggest that marine algal polyphenols and phlorotannins are potential candidates for the suppression of cancer-generating factors.

### 2.7. Anti-Microbial and Antiprotozoal Activity

Infectious diseases caused by fungi, bacteria, and viruses have been a serious threat to public health since ancient times, leading to enormous advances in human medicine [150,151]. With time, microbes have developed their mechanisms to eliminate medicines, resulting in drug-resistant species. At least 50,000 antimicrobial-resistance-related deaths are recorded per year, especially in developed countries, and it is predicted that drug-resistant infections will be responsible for 10 million deaths worldwide by 2050 [151]. Owing to the resistance of pathogens and the lack of effective drugs, a new research area has been opened which aims to explore and develop effective antimicrobial agents with fewer side effects, minimal toxicity, good bioavailability, and better potential compared to the antibiotics currently available in the market [57,151]. The complex secondary metabolites produced by green, brown, and red marine algae can be used to inhibit the activities of fungi, bacteria, viruses, and other microbes [152]. Anantharaman (2002) reported that sterols, halogenated compounds, heterocyclic compounds, and phenolic compounds can be effectively used against both Gram-positive and Gram-negative bacteria [11]. 

Manal et al. (2016) investigated the antimicrobial activities of ethanol, acetone, and hexane extracts of *Padina boryana* (Phaeophyceae) and *Ulva* sp. (*Enteromorpha* sp.) (Chlorophyta) collected from the Red Sea. The results revealed that the ethanolic extracts of *P. boryana* and *Ulva* sp. showed the highest phenolic content among the tested solvents. The results also indicated that *P. boryana* extracts have a higher antimicrobial potential than *Ulva* sp. extracts [153]. The diethyl ether, acetone, ethanol, and methanol extracts of 11 different algal species from the coastal area of Urla, Turkey, were tested for their antimicrobial activity against several pathogenic bacteria under in vitro conditions using the disc diffusion method. Diethyl ether extracts of fresh *Ulva rigida* (Chlorophyta), *Gracilaria gracilis* (Rhodophyta), *Cystoseira mediterranea* (formerly *Cystoseira mediterranea*) (Phaeophyceae), *Ulva lina* (formerly *Enteromorpha linza*) (Chlorophyta), and *Ectocarpus siliculosus* (Phaeophyceae) were effective against all the tested organisms. However, no significant difference in antimicrobial activity was observed between the acetone and methanol extracts of each alga [154]. Several studies have been conducted to identify effective antimicrobial agents against foodborne pathogens. However, it is still possible to use polyphenols extracted from marine organisms to prevent skin-related microbial disease. 

Leishmaniasis is a parasitic disease caused by infection with the parasite Leishmania. The most common forms are cutaneous leishmaniasis, which causes skin ulcers, and visceral leishmaniasis, which affects several internal organs. Ulcers can change in size and appearance over time. Ulcers may begin as papules (bumps) or nodules (bumps) and end in ulcers covered with scabs or crusts. Due to their ineffectiveness and toxicity, the treatments that are currently in use should be replaced [155]. Previous studies noted that terpenes, acetogenins, polyphenols, and alkaloids from algae and other terrestrial plants have been reported to show leishmanicidal activity [156,157]. Several studies evidenced that halogenated terpenoids and acetogenins from genera *Bifurcaria* (Phaeophyceae), *Laurencia* (Rhodophyta), *Dictyota* (Phaeophyceae), and *Canestrocarpus* (Phaeophyceae) featured leishmanicidal and trypanocidal activity [156,158,159,160]. Therefore, we can conclude that algal polyphenolic compounds are a potent bioactive source against skin-related protozoal diseases. 

## 3. Conclusions and Future Prospectives

Algae are one of the most diverse groups of organisms found in marine ecosystems. Naturally, algae are exposed to many stresses, including harmful environmental, chemical, and biological stresses, which have led to the development of several efficient protective systems [120]. Because of this property, they can be considered an invaluable natural resource of bioactive compounds with a wide range of biological activities, such as antioxidant, anti-inflammation, anticancer, anti-aging, whitening, moisturizing, and anti-microbial activities. Owing to their natural origin, low toxicity, and high effectiveness, phenolic compounds extracted from marine algae are promising candidates for the treatment of minor problems, such as skin aging, wrinkling, skin damage including burns and wounds, skin diseases, as well as serious life-threatening diseases, such as cancers [161]. 

There is growing societal awareness on the importance of skin health and integrity to body homeostasis, resulting in a massive demand for effective, eco-friendly dermatological products that delay skin aging and improve skin function and repair capacity. Today, the cosmeceutical industry is among the fastest-growing and most profitable industries worldwide [87]. Therefore, significant development projects and research that aim to introduce these marine algae into the cosmeceutical products are ongoing. This may lead to the development of novel products containing compounds or extracts from natural sources with extraordinary bioactivities that can improve human health and well-being. In addition to evaluating the bioactivities of algae, the availability, affordability, stability, toxicity, and compatibility should be considered to assess the real commercial potential for the industrial production of cosmetics and skincare products [162]. 

**Figure 3:**
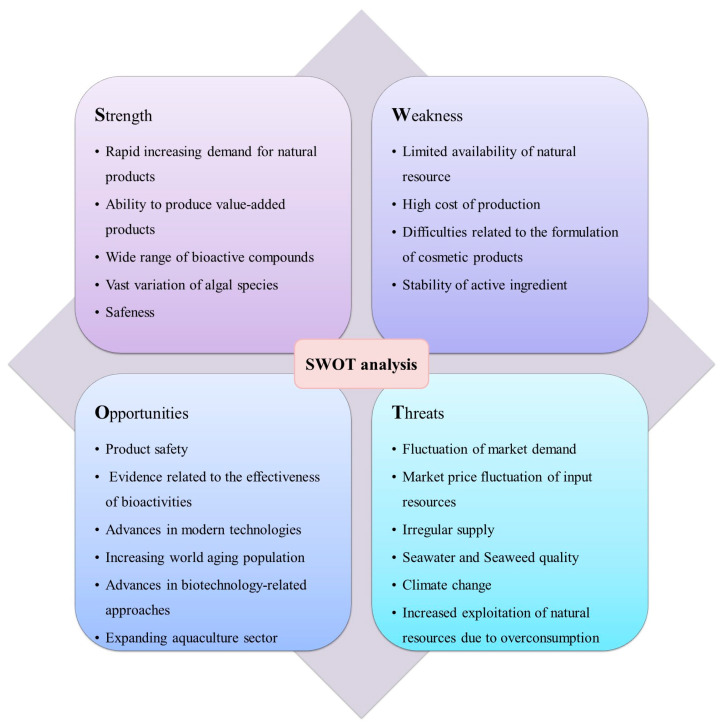
SWOT analysis related to the algae-based cosmeceutical industry.

The demand in the pharmaceutical and cosmetics industries cannot be fulfilled by naturally available algal stocks alone. The development of seaweed-active ingredients depends on the domestication of the respective species. Certainly, it is necessary to understand the biology of algae to ensure a high-quality culture and to increase the content of a compound of interest [163]. Previous studies have generated much interest in the identification of novel ingredients with a wide range of activities for various applications. Advancements in different fields are being explored to expose a broad spectrum of opportunities to develop new products. However, it is essential to ensure the development of functional cosmetics while preserving the properties of active ingredients during the formulation. The cosmetics industry has several strengths and opportunities. However, it has several weaknesses and threats as well [164,165]. Figure 3 illustrates the strengths, weaknesses, opportunities, and threats (SWOT) analysis of the cosmetic industry, which will enable better management of marine algae. By developing strategic tactics, these weaknesses and threats can be minimized. 

Ultimately, the development of marine-algae-containing products will promote physical and psychological well-being in customers by improving their quality of life. In conclusion, this review summarized the potential evidence of the beneficial properties of polyphenolic compounds isolated from marine macroalgae for further development in the cosmeceutical and pharmaceutical industries, combined with consumer demand.

## Data Availability

All data are contained within the manuscript.
